# Exploring How Substance Use Impedes Engagement along the HIV Care Continuum: A Qualitative Study

**DOI:** 10.3389/fpubh.2016.00062

**Published:** 2016-04-08

**Authors:** Marya Gwadz, Rebecca de Guzman, Robert Freeman, Alexandra Kutnick, Elizabeth Silverman, Noelle R. Leonard, Amanda Spring Ritchie, Corinne Muñoz-Plaza, Nadim Salomon, Hannah Wolfe, Christopher Hilliard, Charles M. Cleland, Sylvie Honig

**Affiliations:** ^1^New York University College of Nursing, New York, NY, USA; ^2^Center for Drug Use and HIV Research, New York, NY, USA; ^3^Peter Krueger Center for Immunological Disorders, Mount Sinai Beth Israel, New York, NY, USA; ^4^Mount Sinai St. Luke’s-Roosevelt Hospital Center, Spencer Cox Center for Health, New York, NY, USA

**Keywords:** HIV care continuum, antiretroviral therapy, barriers, qualitative, drug use, African-American, Black, Hispanic

## Abstract

Drug use is associated with low uptake of HIV antiretroviral therapy (ART), an under-studied step in the HIV care continuum, and insufficient engagement in HIV primary care. However, the specific underlying mechanisms by which drug use impedes these HIV health outcomes are poorly understood. The present qualitative study addresses this gap in the literature, focusing on African-American/Black and Hispanic persons living with HIV (PLWH) who had delayed, declined, or discontinued ART and who also were generally poorly engaged in health care. Participants (*N* = 37) were purposively sampled from a larger study for maximum variation on HIV indices. They engaged in 1–2 h audio-recorded in-depth semi-structured interviews on HIV histories guided by a multilevel social-cognitive theory. Transcripts were analyzed using a systematic content analysis approach. Consistent with the existing literature, heavy substance use, but not casual or social use, impeded ART uptake, mainly by undermining confidence in medication management abilities and triggering depression. The confluence of African-American/Black or Hispanic race/ethnicity, poverty, and drug use was associated with high levels of perceived stigma and inferior treatment in health-care settings compared to their peers. Furthermore, providers were described as frequently assuming participants were selling their medications to buy drugs, which strained provider–patient relationships. High levels of medical distrust, common in this population, created fears of ART and of negative interactions between street drugs and ART, but participants could not easily discuss this concern with health-care providers. Barriers to ART initiation and HIV care were embedded in other structural- and social-level challenges, which disproportionately affect low-income African-American/Black and Hispanic PLWH (e.g., homelessness, violence). Yet, HIV management was cyclical. In collaboration with trusted providers and ancillary staff, participants commonly reduced substance use and initiated or reinitiated ART. The present study highlights a number of addressable barriers to ART initiation and engagement in HIV care for this vulnerable population, as well as gaps in current practice and potential junctures for intervention efforts.

## Introduction

The tolerability, dosing schedules, and efficacy of antiretroviral therapy (ART) for HIV infection have improved substantially in recent years, allowing persons living with HIV (PLWH) to live longer lives, and experience a higher quality of life, than in the earlier phases of the epidemic (1). Yet, in the United States (U.S.), there are serious gaps in rates of engagement in HIV care, uptake of ART, and clinical status – a series of stages referred to as the “HIV care continuum” (2). Specifically, of the 1.2 million PLWH in the U.S., an estimated 60% are not well retained in care, more than half are not prescribed ART, and only 25% have undetectable viral loads, the ultimate goal of HIV care, with equally poor rates of engagement found among the population of men who have sex with men (MSM), who comprise approximately half of all PLWH, and other subgroups of PLWH (3). However, African-Americans/Blacks and Hispanics have lower rates of engagement along the HIV care continuum compared to Whites, and in response, the Centers for Disease Control and Prevention (CDC) has called for efforts to reduce these racial/ethnic disparities (4).

Overall, rates of engagement in HIV care, ART initiation, and adherence to ART are less than optimal for persons who use illicit drugs or use drugs non-medically and non-users (5, 6). However, those who use illicit drugs have higher rates of morbidity and mortality compared with their HIV-infected peers who do not use drugs (7–9). Moreover, at the individual level, substance use problems are associated with decreased access to health care, less frequent engagement in HIV primary care, and reduced likelihood of being prescribed ART (5, 7–9). Indeed, while the literature documenting the associations among alcohol and drug use and poor engagement along the HIV care continuum is substantial (5), and there is a consensus that HIV and substance use are twin epidemics (10, 11), the mechanisms that underlie these associations remain poorly understood. Past research has found that drug use is commonly used as a coping strategy when HIV is first diagnosed (12) and can impede engagement in care – a critical early stage of the HIV care continuum. Furthermore, qualitative research has found that PLWH who internalize stigma related to both HIV and substance use are at grave risk for depression (13), a known barrier to good HIV outcomes. Wilson and colleagues reviewed the qualitative research literature pertaining to African-American/Black MSM. The review highlighted the importance of layered stigma and understanding health behavior, including substance use, in context, as well as evidence of resilience in this heterogeneous population (14). The present study extends this emerging literature using a multilevel level theory to potentially uncover and explore mechanisms and processes by which substance use interferes with engagement along the HIV care continuum, at the individual-, social-, and structural-levels of influence, and how barriers can be overcome, from the perspectives of an under-studied population at high-risk for poor outcomes: low-income African-American/Black and Hispanic PLWH who use/have used drugs, and who have delayed, declined, or discontinued ART, including sexual minorities.

We explore how substance use impedes engagement in the HIV care continuum through a version of the Theory of Triadic Influence (15). The Theory of Triadic Influence is a multilevel social-cognitive theory focused on three “streams of influence” that act simultaneously to affect health behaviors: the individual level (e.g., poor knowledge about ART and HIV care; health beliefs such as medical distrust; negative emotions, particularly fear of ART; mental health and substance use problems), social level (e.g., unfavorable peer norms that impede HIV care and ART, HIV, and other stigmas, relations with providers), and structural level of influence (e.g., aspects of health-care settings including their location, and the ability to access ancillary substance use treatment and mental health care). Thus, the Theory of Triadic Influence is useful in three respects: first, it guides the selection of domains to explore; it draws attention away from a sole focus on individual-level barriers, which are perhaps the most visible, but not necessarily the most potent or important; and as a social-cognitive theory, it highlights the interplay of the multilevel factors that influence behavior.

For African-American/Black and Hispanic communities facing multiple and competing challenges to their health and well-being, which stem in large measure from disproportionately high rates of poverty, ART initiation and engagement in HIV care are especially complex processes. Moreover, these barriers to ART and HIV care are grounded in a historical and cultural context that includes past maltreatment of populations of color in medical research, with the Tuskegee Syphilis Study serving as the most notorious example of such abuses, which resonate with present-day exclusion, structural inequality, and discrimination (16, 17). Thus, this historical/cultural context may serve a filter through which HIV care and ART, including the side effects of ART, are experienced.

Importantly, the literature on the HIV care continuum focuses heavily on whether PLWH are prescribed ART, and rates of viral suppression, but there is almost no attention to the intermediate stage of individuals electing to initiate ART (18, 19). The present study addresses that gap in the literature. We also wish to note that the problems of poor engagement along the HIV care continuum are related. For example, PLWH who have declined ART also tend to avoid HIV care, because they do not wish to discuss or fear being pressured to take ART, or because they do not see the need for HIV care if not on ART (20–22); and those not well engaged in HIV care rarely gain access to ART (23). The present study attends to ART initiation and engagement in HIV care as two separate but related stages in the HIV care continuum.

### Aims

The aims of the present study were to examine the experiences of an at-risk population: African-American/Black and Hispanic PLWH who have delayed, declined, or discontinued ART, many of whom also insufficiently engaged in HIV primary care. The study explores how substance use serves as a barrier to engagement along the HIV care continuum through its influences on factors, such as access to services, social norms and stigmas, attitudes toward and emotions regarding ART and HIV care, and personal decisions. The qualitative research approach is appropriate for examination of multifaceted, multilevel phenomena such as these (12). We hypothesized that the individual perspectives and lived experiences of African-American/Black and Hispanic PLWH would reveal both the complex social realities that undermine their uptake of ART and engagement in HIV care, the meaning of ART and HIV care, and illustrate the diverse ways they have navigated these challenges, with varying degrees of success. Thus, qualitative studies of this kind are crucial to address the individual, social, and structural contexts that either limit or promote African-American/Black and Hispanic PLWH’s access to, and use of, life-saving care and treatment.

## Materials and Methods

### Recruitment

Participants were adult African-American/Black and Hispanic PLWH (*N* = 37) recruited through peer-to-peer referral and in hospital-based HIV clinics who were medically eligible for (based on national guidelines at the time) but not taking ART, as part of a larger intervention development study on increasing ART initiation in this group. Participants were purposively sampled from a larger sample of 95 PLWH approximately 4–6 months after enrolling into the main study. The sub-cohort of 37 PLWH was selected for maximum variation with respect to past experiences with ART and length of time living with HIV. Almost all participants in the larger study reported past substance use (>85%) and/or substance use problems, and about half reported recent substance use, largely non-injection drug use. Thus, we did not sample participants for this qualitative sub-study on substance use criteria, and operated under the assumption that, given the prevalence of substance use in the lives of PLWH, both past and present substance users and non-users would have perspectives on how substance use may interfere with HIV care and ART initiation, continuation, and adherence. The larger study, its recruitment methods, and characteristics of the larger sample are described elsewhere (24). Procedures were approved by the Institutional Review Boards at New York University and two collaborating hospital sites.

### The Local Context

New York City (NYC) has a large and mature HIV epidemic, with approximately 115,000 PLWH, more than 75% of whom are African-American/Black and Hispanic (25). Overall, NYC has achieved higher rates of engagement along the HIV care continuum than among PLWH nationally [e.g., 41% in NYC are virally suppressed compared to 25% nationally (26, 27)]. The city hosts a large network of HIV care facilities and support services, including Designated AIDS Centers and AIDS service organizations, and some PLWH receive services in local general community-based organizations and from private facilities (28). Furthermore, all patients in NYC have access to ART at low or no cost (29). Nonetheless, NYC evidences serious gaps along the HIV care continuum, and many PLWH are diagnosed late in the course of their HIV disease (30). Racial/ethnic disparities in engagement in care, ART initiation, and viral suppression are similar to national patterns, where African-American/Black and Hispanic PLWH show the lowest rates of engagement along the continuum (31). Moreover, HIV-related death rates are concentrated in the highest-poverty neighborhoods (25). Last, quality of HIV care varies within care settings [for example, where opioid users receive lower quality care than non-users (32) and across settings (33)].

### Procedures

Data collection took place in 2012–2013. Trained, experienced research staff conducted in-depth semi-structured interviews until data saturation was reached on core constructs (34), as determined by a senior qualitative researcher, who reviewed transcripts and queried interviewers during data collection. Participants gave signed informed consent before engaging in study activities. Interviews were audio-recorded and professionally transcribed verbatim for analysis. Interviews were conducted in-person in a confidential location, and each lasted 60–90 min. The interview questions followed a guide with core questions and prompts that were grounded in the domains of Theory of Triadic Influence, covering specific topics relating to (a) the history of participants’ personal decisions about ART initiation and continuation or decline/discontinuation, (b) past experiences with ART (where appropriate), (c) perspectives on factors that impede or facilitate access to ART, probing for individual, social, and structural levels of influence, (d) attitudes toward, and experiences with, HIV primary care, and (e) emergent themes. The role of substance use was explored in each of these topic areas.

### Data Analysis

A systematic content analysis approach was used in phases to identify processes and mechanisms of action in the interview transcripts (35, 36). Data were analyzed in the Dedoose platform. First, the research team developed a set of preliminary descriptive codes based on an initial review of the transcripts and the domains of the theoretical model (e.g., emotional barriers, relevance of race/ethnicity, social class, sexual minority status, and/or gender, relationships with providers, peer norms). Then, a trained qualitative analyst coded each interview transcript using these codes, refining and creating additional codes when necessary. We conducted a reliability check of the coding in which 25% of the interview transcripts were independently coded by a second qualitative analyst to establish high inter-coder reliability and further refine the existing set of codes (35). Discrepancies in coding were resolved by consensus. We used the Memo and Code Cooccurrence functions within Dedoose to support analysis of relationships between codes and develop larger themes. The two qualitative analysts together then identified areas of congruence and discrepancy with respect to the broader themes. Finally, excerpts were selected to illustrate the main findings and to expand upon the selected themes.

## Results

### Participant Characteristics

Participants ranged in age from 26 to 64 years (M = 48.7 years, SD = 9.37 years), as shown in Table 1. The majority (59.46%) was male, and among the male participants, 59.10% identified as gay, bisexual, or non-heterosexual. Most (78.38%) were African-American/Black and 21.62% were Hispanic. Nearly, all (97.3%) were from lower socioeconomic status backgrounds. On average, participants had lived with HIV for 13.88 years (SD = 8.11 years). Approximately half (54.05%) had taken ART at least once in the past, and on average, had initiated ART on 5.17 different occasions (SD = 5.44 times) prior to study enrollment. None were on ART at the time they enrolled in the study. Most (72.97%) rated their health as “good” or better, and the majority (83.78%) had an established health-care provider in the past year, mainly at a hospital-based HIV clinic (77.78%). HIV health indicators highlighted the need for ART based on relatively low CD4 counts, also called T-cells [average CD4 = 291.87 cells/mm^3^ (SD = 144.15 cells/mm^3^)] and substantial HIV viral load levels [mean log_10_ HIV viral load 3.08 (SD = 1.36)], the primary indices upon which HIV treatment decisions are based (37). All reported that a health-care provider had recommended ART in the past. Half (50.00%) had symptoms of depression at a clinically significant level. Regarding substance use, daily tobacco use was common (67.57%), and half (54.05%) had used illicit drugs in the past 6 months, such as marijuana, cocaine, crack, and heroin. Almost one-third (29.73%) used drugs daily in the past 6 months. Approximately 10% (10.81%) drank alcohol at least four times a week in the past 6 months, and nearly one-third had participated in substance use treatment in the past 6 months (30.56%). A history of injection drug use was common (40.54%), and 2.7% had injected in the recent period. Furthermore, 8.11% were enrolled in a methadone maintenance treatment program (MMTP) in the past 6 months. Thus, participants were diverse with respect to sociodemographic, health, and other background characteristics, in keeping with the sampling approach. A description of the psychometric characteristics of structured measures used to assess the sample is described elsewhere (24).

**Table 1:** **Description of the sample (*N* = 37)**.

	Mean (SD) or %
Age (years)	48.7 (9.37)
Male sex	59.46
If male, identifies as gay, bisexual, or non-heterosexual	59.10
African-American/Black, not Hispanic	78.38
Hispanic/Hispanic	21.62
Low socioeconomic status	97.3
**Health indicators**	
Years since HIV diagnosis	13.88 (8.11)
Ever taken ART in the past	54.05
Number of times started/stopped ART	5.17 (5.44)
Health self-rating “good” or better	72.97
Had an established HIV care provider over the past year (MRF)	83.78
Receives care in hospital-based HIV clinic	77.78
Average CD4 in the year before baseline (MRF)	291.87 (144.15)
Average log_10_ viral load in the year before baseline (MRF)	3.08 (1.36)
Health-care provider recommended ART (lifetime)	100
Depression screener at a clinically significant level (CES-D8)	50.00
**Substance use**	
Smoked cigarettes daily past 6 months	67.57
Any drug use past 6 months	54.05
Daily drug use past 6 months	29.73
Alcohol use 4+ times a week past 6 months	10.81
Substance use treatment – past 6 months	30.56
Ever injected drugs (lifetime)	40.54
Injected drugs in the past 6 months	2.7
Enrolled in methadone maintenance treatment program (MMTP) in the past 6 months	8.11

### Qualitative Findings

#### Overview

Study findings illuminated relationships among ART uptake, HIV care, and substance use for African-American/Black and Hispanic PLWH. Overall, decisions regarding and experiences with ART were highly complex and emotionally charged, while discussions of HIV care attendance patterns were less significant. For this reason, the majority of the themes described below pertain to ART initiation, while engagement in HIV care is either not explicitly discussed or is implied. Participants’ ranges of experiences can be broadly organized into the following domains. First, heavy substance use, but not necessarily casual or social substance use, impedes ART uptake by reducing available psychological resources to manage medications, and by triggering depression, which reduces motivation to maintain health behaviors. Yet, management of one’s HIV infection is not a simple linear event, and changes to “lifestyle,” followed by initiation or reinitiation of ART were common. Second, barriers to ART are embedded in the other challenges that PLWH face in their lives, which disproportionately affect poor African-American/Black and Hispanic PLWH of color (38, 39). Third, some participants experienced stigma in HIV medical settings and from other institutions, mainly associated with their substance use or past substance use. Last, in collaboration with trusted providers and ancillary staff, it was common for participants to reduce their substance use, access needed psychosocial services, reengage in HIV care, and, in some cases, initiate or reinitiate ART. All names used below are pseudonyms, and some identifying details have been changed.

#### Mechanisms by Which Heavy Substance Use Impedes ART Uptake

Consistent with the literature, participants reported heavy substance use, particularly crack cocaine use, can indeed interfere with ART adherence, and this, in turn, reduced motivation to initiate or reinitiate ART. Luzio, a 49-year-old sexual minority man of mixed race (White/African-American), who was diagnosed over 25 years ago, described how substance use can interfere with ART:

(Substance use) does (interfere) at times. Because generally the crack, it kind of demands full attention when I am doing it. Yeah. I am not too functional. I could have the medication right there, and while doing crack say, oh, I don’t feel like taking them now, keep putting them off and time seems to fly when you are doing it, that is one major thing. At times I plan to take them at noon, but then you have to take them with food, and when I am doing the crack I don’t have any appetite whatsoever, so it becomes non-conducive to take the meds. [112030]

Moreover, participants’ accounts provided further insight into how heavy substance use can interfere with HIV medications. LaTonyia is a 54-year-old heterosexual African-American female who was diagnosed with HIV approximately 10 years ago. She described how fatigue, depression, and related symptoms, which typically followed periods of heavy substance use, left her depleted and unable to manage the demands of medical appointments and/or adhere to her medication regimen, a pattern echoed by other participants:

Well for me, I don’t tend to take my medications when I’m using drugs … ‘Cause I get lazy, or I’ll get depressed, I wanna sleep all day, I don’t wanna take my medicine … It becomes a major part of a problem when you usin’ drugs, for me, takin’ HIV meds. But I just don’t care. It’s difficult. [511031]

Furthermore, PLWH, and in particular, African-American/Black and Hispanic individuals who often have high levels of medical distrust, as discussed above, tend to fear ART side effects (22), and these fears were heightened for substance-using PLWH. As explained by Bill, a 64-year-old heterosexual Latino man who was diagnosed over 25 years ago, uncertainty about the potential interactions between street drug and ART increased feelings of apprehension about beginning or continuing ART. In the following quote, Bill outlines the potential dangers that could result from mixing street drugs and ART:

Street drugs has a lot of shit in there that shouldn’t be in there, you know what I mean? So I do know that some of the medications (give you) nightmares. Some medication has some strong effects to them, you know? So you know there could be a combination, you know? They could be something that’s not going to click, you know what I mean? And you don’t know, they could put something in the crack that your HIV meds won’t attract to, you know what I mean? ‘Cause a lot of shit going through street drugs, you know what I mean? And they are some HIV medications that don’t react properly [512057]

Yet, taking ART is generally expected of PLWH, by their families, peers, and providers, and substance use is considered unacceptable for PLWH who “should” be on ART. Thus, participants reported finding it quite difficult to raise these concerns about possible street drug–ART interactions with their health-care providers. This is complicated by the fact that participants commonly feel “pressured” to take ART, and that providers are heavily invested in “getting” the patient to take ART, rather than, as participants would like, considering “the whole person,” their individuality, their life contexts, and the complexities of HIV and ART. Jonsie is a 40-year-old heterosexual Latina diagnosed approximately 10 years ago, who has never taken ART. Jonsie describes these tensions that emerge at the interface of health-care providers who provide access to life-saving medications and who treat a complex health condition in the context of a short encounter, and patients who have strong, complicated feelings about ART, which cannot necessarily be separated from feelings of distrust of medical institutions and medications that are common in communities of color:

It’s pressure because they tell you if you don’t take your medication what can happen to you. But at the same time, they don’t tell you when you take your medication what’s gonna happen to you too (side effects, toxicities). And when you do ask the doctor (about long term side effects), the doctor tell you oh, don’t worry about that right now. Right now just what you gotta worry about now because your – your T-cells 200 – I mean your viral is 200,000. This is what you better worry about right now. So in my mind I’m like, well, I’m not sick right now so then why even bother. [511037]

Jonsie’s narrative highlights a common misconception held by PLWH: that ART can be delayed when CD4 counts are low if the individual does not feel sick. Yet, these types of misconceptions, which often have emotional and cultural roots, are not easily addressed in a brief medical encounter by a provider who is appropriately focused on treating a complex medical condition. Thus, importantly, Jonsie highlights a paradox many PLWH find themselves in: ART may improve CD4 counts and reduce viral load, but ART can bring serious long-term toxicities. While only a serious health crisis may break this deadlock for some PLWH, delaying ART until CD4 counts are very low can cause permanent damage to the immune system (40).

Although many participants viewed their substance use, particularly heavy use, as incompatible with ART and engagement in HIV care, some were able to adopt strategies to manage their substance use behaviors while taking ART with good adherence. Walter, a 32-year-old African-American sexual minority man, diagnosed within the past 5 years, shares an earlier time when he was reluctant to begin an ART medication regimen. He outlines his concern that beginning ART would require him to make dramatic lifestyle changes to his recreational drug and alcohol use, thus potentially interfering in the clubbing and other group activities he takes part in with his peers, activities that are meaningful to him as a young Black gay man and that affirm his belonging to a community. As Walter explains:

Well, I didn’t want to keep popping pills every day … And it’s not a fact of (being a) reminder of me being a positive, but it was just the fact that I have to make sure to remind myself that once I start (ART) I can’t stop … And I have to keep going and keep going and keep going. I knew that some things about my lifestyle would have to change. [212023]

But at the time of his interview, Walter had recently begun taking ART and reported a fairly seamless introduction to integrating medication into his life. In elaborating upon his decision-making process, Walter describes how he found a way to address his concerns with the assistance of his HIV/AIDS providers and ancillary staff. The personalized information and other guidance he received enabled him to adapt his drug and alcohol use activities to the demands of his regimen, meeting both his medical and social needs:

(T)hat was kind of my main concerns was like, okay, how much intake I was going to have to do when it came to alcohol, my marijuana intake … So actually those concerns and questions were actually being answered … I did talk to them about like the marijuana intake and my alcohol intake and they were very helpful with giving me information and letting me know that there are some studies that you can take your medication with alcohol. I was just told from my doctor yesterday, she said if you are going to drink, drink at least a couple hours before you take it. So if I’m going out drinking between like the hours of 8 or 9, and that’s usually when I take my medication is like 9 or 10. She said wait about at least three to four hours after you’ve had your alcohol and then take your medication. [212023]

Thus, Walter recognized that changes to the frequency, timing, and/or quantity of substance use would be warranted before initiating ART, but these changes may be realistic when motivation to start ART is high, and support services, including harm reduction services, are in place.

#### Barriers to ART Initiation Are Embedded in Other Challenges That Low-Income PLWH Face

Rita is a 54-year-old heterosexual Latina diagnosed 15 years ago who provided a narrative below that reveals the vulnerabilities, uncertainties, and even chaos pervasive in the lives of study participants:

I started (ART) in the 80s, I was on AZT and I stopped. And then in ‘96 I was on Combivir and something else. And then like in 2004 I think I was on some other meds … When it [Norvir] first came out it was a liquid and my daughter would help me take it … (S)he would put the medicine in, and then put on the peanut butter. That’s how we got my T-cell count to go up. But then I stopped, I started doing drugs again. Then I moved, and I was in an abusive relationship, and I had to leave my daughter and everybody, and I had to move. And then I got on (housing for people living with HIV). And, I started taking (HIV) medicine again and then I stopped again when I was homeless again. I’ve just been through a lot. [511042]

Thus, Rita’s cycles of ART discontinuation and reinitiation followed a tortuous path through her periods of homelessness, the burdens of drug use, an abusive relationship, and as she describes elsewhere in the interview, intense depression and forced separation from her daughter. Rita’s alternating moves toward and away from ART cannot be disconnected from the layers of her life that she narrates, and through which she has made sense of her relationship to her HIV infection, and to HIV care and ART. Indeed, the problem of discontinuing ART is entwined with the larger context of poverty and its consequences. And yet, by the time of her interview, Rita had significantly reduced her drug use, reestablished care with a provider, and reinitiated ART. In fact, Rita had been taking a once-a-day ART regimen for about 2 months at the time of her interview. When asked what had enabled her to reinitiate and adhere well to an ART regimen, Rita described the “simple things,” that helped her, such as housing, access to juice, and a helpful partner also living with HIV and taking ART:

Yeah, I’m good with it, I’m taking it every day, I don’t miss no doses … I don’t like doing it. Trust me, I don’t like it. I don’t like having to remember to do it. But it’s right in my bed stand and it’s a habit now. I tell my boyfriend to go get my juice. That’s how I take my medicine, he takes his and I take mine … If I tell him to get my juice to take my medicine then it reminds him to take his medicine. And if his bottle’s there then I know I have to take my medicine. It’s the simplest things. [511042]

Rita’s characterization of these supports as “simple” reveals how important ordinariness can be to the management of HIV, particularly when one has experienced so much chaos in the battle to survive homelessness, substance use, intimate partner violence, poverty, and HIV simultaneously. Moreover, she highlights the critical role of stable housing and social support in ART management.

#### Perceived Stigma Is a Major Barrier to ART and HIV Care

Participants reported mixed experiences in health-care settings. Both supportive and trusting relationships and negative interactions with providers were commonly reported, as we describe in more detail below. In part, this may be due to variability in the type and quality of settings that PLWH encounter, as well as to the large number of social service and health-care providers they encounter in the course of managing a complex health condition and cooccurring conditions (41). Thus, some settings may enact policies or procedures that, perhaps inadvertently, result in stigma, and some providers may behave in a manner that stigmatizes PLWH, particularly those who use or who have used substances (42, 43). It is also possible that PLWH’s position in a group vulnerable to “structural violence,” namely, when social structures or social institutions harm individual by preventing them from meeting their basic needs (44), increases their awareness of, and sensitivity to stigma or perceived stigma (45). In particular, participants described ways in which their past or current substance use, or the perception among providers that they use or have used substances, shaped the quality of and their access to HIV care and ART initiation.

In the following quote, Martha, a 49-year-old heterosexual Latina diagnosed 15 years ago, discusses the judgment she experiences as coming from one of her health-care providers:

So this provider she really gets on my nerve and she’s a physician assistant. I don’t think she know a damn thing about HIV meds and she just does whatever … And judge me … Alright, so what? I used drugs and I was a prostitute. And I don’t always use a condom … alright, alright, and …? [511038]

Martha’s perceptions of stigma and judgment in the health-care setting are not limited to clinic staff, but extend to other institutions as well. Martha described how her parole requirements, as far as she understood them, had left her with little choice but to comply with her ART regimen, lest she be sent back to prison:

I was on parole … (T)hey did threaten me if we discover you’re not taking your medication we’re going to lock your ass up. Straight like that, so that was kind of an incentive. Because they were going to come and count your pills. They never did it, but I don’t know. They could have come to the house and said get your pills. We counting. Put on a glove. The same way they come and do the urine, you know. [511038]

While good adherence to ART has marked health benefits, coercing ART adherence may have the counter-productive effect of undermining one’s intrinsic motivation to take ART (46). In fact, Martha did discontinue taking ART, but is also troubled by her declining health. She is overwhelmed by the pressures and conflicts she faces from many different directions: the demands of her daughter and granddaughter who are currently homeless and staying with her in her small apartment; the pressures she encounters from her HIV-positive peers, who suggest she is “stupid and could die” if she does not take ART; and a recent break-up that has left her emotionally reeling. In addition, the judgment she perceives from health-care providers, and the difficulty involved in finding a new one, have compounded Martha’s stress and led her to become withdrawn and isolated. Consequently, she is coping with these multiple stressors by putting off the decision about reinitiating ART, but is still gripped with the growing fear of contracting an opportunistic infection, a decline in health, or even dying during this period before she is ready to restart ART:

So at some point I’ve got to get on track. I don’t want to be beating myself in the head with it and then now all of a sudden I’m sick, you know. Worrying and being stressed. I do that on my own because I’m like to myself, damn, I’ve got to take my medication … And I don’t have the best provider on the earth. But it’s stressful to try to find a provider. For me, it’s stressful for me. [511038]

Franklin is a 64-year-old heterosexual African-American man diagnosed 20 years ago. Like Martha, Franklin also experiences judgment from his health-care provider. In the following excerpt, Franklin discusses his frustration with the poorer quality of care that he receives, in contrast to a more privileged patient in the same setting:

I got pissed behind that one time because – at that particular time I was using (drugs), okay. (The doctor) knew I was using because I was coming up with the positive urines … And this guy walked in all nice and clean and – and I seen the doctor come out and (he) said, excuse me Franklin, I just wanna speak to this gentleman for a second. I said yeah, but – what do you mean? I gotta leave too. He say, listen, just give me five minutes. You know he took the guy in the office. They was in there for almost an hour. And then he comes back out and apologize to me, I’m sorry it took so long. Why, because the guy had a suit and tie on. This was the impression he gave me … And he could look at him and tell that he must have had a good job but he’s HIV, but he had priority over me because the class of person that he was in. [112014]

Thus, Franklin expresses his concern to his doctor (“I gotta leave too”), only to be met with the promise of, “just five minutes.” The doctor’s subsequent actions illustrate what his reply actually translated to, the simple but powerful directive: “wait.” Unlike the freedom to speak and welcome which the doctor extended to the well-dressed, unscheduled patient, Franklin felt little choice but to wait for his own appointment and grapple with the implications of being a poor, African-American, HIV-positive, and drug-using man whose time is treated as less valuable than that of his wealthier peers – a hierarchy created and reinforced, in many cases, by the larger society.

Whereas Franklin’s experience represents the differential care that substance-using poor African-American/Black and Hispanic PLWH of color may encounter through a particular incident, Chryssy’s narrative speaks to the larger negative perceptions she believes are held by some providers toward their patients, and by the larger society, a phenomenon that she, a 49-year-old heterosexual Latina diagnosed 5 years ago, sees as a mundane, even commonplace, aspect of her and her HIV-positive peers’ experiences.

Most of everybody that I know has HIV, because I live in an HIV community and like to describe it as a quagmire. You get cooled down into this – the lowest form of life because we all live below the poverty level, we don’t have jobs, and it is not because we are not qualified to have jobs, but because it is not practical to have jobs (because you will lose your public assistance benefits). And the outside world, the mental health providers, and the other people who provide services to you often mistreat you. [512038]

Chryssy also discusses how she was greatly affected by the death of her life partner two decades earlier, due in part to kidney failure following experimental treatment for *Cytomegalovirus* (CMV). The feelings of grief and loss continue to affect Chryssy daily, and she had a period of intense drug use immediately following her partner’s death. Furthermore, she faces suspicion from providers that she had sold her ART medications, presumably to buy drugs:

Oh, okay, let me ask you this question, blah blah blah. Well did you sell your medication? Automatically that is the first question, did you sell them? No I didn’t. I am only going to tell them this and this and that … I say I am not taking medication, but the conclusion that they will jump to is that, oh you just want to sell your medication, you know? People are very complicated. Like, people’s lives are very complicated and we cannot all be put into a particular box as to why we do what we do. [512038]

Participants describe that in NYC, as in other major urban areas, there exists a thriving underground prescription drug economy for the resale of ART, mainly to pharmacies, often called “drug diversion” (47). In fact, some participants had sold their ART prescriptions in exchange for cash, almost always to corrupt pharmacies, whereas many others had heard about or were familiar with these practices. In the following quote, Ari, a 64-year-old heterosexual African-American male, diagnosed over 20 years ago but who has not yet taken ART, shares his observations of his HIV-positive peers who sell their medications to pharmacists for “easy money,” motivated by their desire or need for illicit drugs, and, he believes, with little regard for their own health:

You know everyone that I know that sells their medications, they know the consequences of not taking that medication …. And if you have a history of using drugs and if you actively using now you’re definitely gonna through the measures of trying to get everything – do everything that’s possibly to get money. Medication is the … easy way of getting money, okay, and they know the consequences of not taking medication. They know their T-cells are dropping, their immune system is weak and they don’t care. They’re willing to sacrifice that. [112014]

However, not all participants would agree with Ari’s point that the HIV-positive person who sells their medication in exchange for cash (and as some reported, sundries such as toothpaste and deodorant) is fully cognizant of and accepts the known risks of not taking their medications. While undoubtedly some are, others may not fully understand its effects, nor would they have the opportunity to discuss these concerns with their prescribing doctors, as to do so would threaten their relationships with providers and prevent their involvement with this informal cash economy. Luli is a 50-year-old Latina sexual minority diagnosed 8 years ago. According to Luli, her doctor’s ambivalence as to whether she should begin medication or not was evidence that she could manage without them, and she does not expect that her decision to sell her ART medications will adversely affect her health. Moreover, Luli views that her decision to delay ART had another significant benefit, a financial one:

And I know I think I’m killing myself but until the doctor say I’m killing myself, I’m not killing myself. It’s like the devil on this side saying do it, do it, do it. Doctor said I should take it but then she said I don’t need it. That’s when I said, well, if I don’t need it I’m not going to take it. But then there’s money in it … It was worth it for me. I’m going to be honest with you. He [the pharmacist] take the med, so I get $200. My numbers is not being damaged further and then it’s not like I’m taking the money and just fucking it up. For me to get the extra money, because we set on a set budget. And sometimes when you have $330 (a month), an extra $200, I could buy some nice stuff. [511033]

Implicit in Ari and Luli’s narratives, and underlying the need or desire to divert ART for cash, are the constraints of poverty and difficulties surviving on public assistance, and, perhaps, unmet need for harm reduction and other acceptable substance use treatment services. As Luli suggests, funds from drug diversion nearly double her monthly income – and this may be difficult if not impossible to resist when offered by the pharmacy, including in geographical locations with a high cost of living but relatively modest monthly government entitlements. As noted above, the problem of ART delay or discontinuation cannot be separated from the larger context of poverty.

#### Positive Relationships with Health-Care Providers Facilitate ART Uptake and HIV Care

For the majority of participants, the ability to communicate openly and honestly with health-care providers played a critical role in determining whether they were willing to continue to seek HIV care and to eventually consider initiating or reinitiating ART. Individuals reported being substantially more forthcoming with their providers when they perceived them as non-judgmental and compassionate, and when they felt that providers gave them the time and information necessary to come to an independent decision about ART. Being able to speak openly about substance use and how this relates to an individual’s readiness to initiate ART on one’s own terms were also repeatedly invoked as positive aspects of participants’ HIV care experiences, and in turn as important facilitators to continuing to seek HIV care and to consider initiating ART.

Luzio describes how he recently recommitted to an ART regimen, following several years of poor adherence and stopping and starting ART. Since the passing of his lover and his drug relapse several years prior, Luzio’s goal is not to attain full sobriety as he had done in the past, but to manage his drug use in preparation to restart his regimen. This process included, as he put it, “telling the truth” to his provider regarding his inconsistent use of ART and disclosing that he had been selling the medications he had been prescribed. Luzio recalls that he had shared similar information with other doctors previously, who he said “really gave it to him,” to which he responded by disengaging from care. How did he know that this disclosure process would be different with his current doctor? Luzio explains that these doctors do not judge or condemn him, and as he expected, listened to him, gave him support, and helped him plan and prepare his reinitiation of ART:

Actually my provider, my primary is on maternity leave and before she left I told her I planned on starting (ART). She did urge me, she said you need to get off those drugs, I mean not preachy like or anything, not in a way that I was apprehensive or anything, but get off those drugs and get onto your meds. She said, I am going to go on maternity leave probably for a couple of months and I hope to see you have begun your meds. Which I have, so she has been gone about three weeks and I have started like a week after she – but I saw in lieu of her, because she is on leave, a doctor I seen before, used to see actually, from the same clinic. Yeah. He is encouraging too. [112030]

Mental health care is another critical aspect of ART initiation. Dewan is a 53-year-old heterosexual African-American man, diagnosed within the past 10 years, who was considering initiating ART, and who did eventually decide to start taking it. He discusses the support he received from his therapist to address the problems substance use were causing in his life, which included, but were not limited to, reinitiating ART:

She was very helpful. She constantly called me at my home, at that time I was battling a severe substance abuse problem, y’know what I mean? And I would actually talk to her about that. And she actually helped me get sober. She really did, yeah [name deleted]. Yup, mm-hmm … I remember she would call me down for a meeting and I would go in there bawling and crying. And I could talk to her about anything. There was nothing I couldn’t talk to her about. ‘Cause she knew that, one thing she did say to me, she said part of getting you to take medication, and getting ready to make this decision is that you got all this other stuff, y’know, on the fire, y’know what I mean, how it’s hard for you to really even consider to take this medication. [211009]

Dewan’s therapist facilitated open communication about a challenging issue and allowed him to address his substance use issues and helped move him forward in his goals to take ART. By contrast, Dewan’s experiences with his medical providers were markedly different from that with his therapist. Below, Dewan discusses how providers assumed that he would never take medications, even though he had expressed wanting to do so for some time:

First of all, nobody could believe I actually took the medicine. And see that’s what bothered me. That God, you guys thought that little of me, y’know what I mean, your opinions? [voice changes to one of surprise] “Oh shit, you actually takin’ it?” Like, what you think? I didn’t want to take it? That means, it sounded to me like nobody listened. Nobody really listened to “Dewan.” Y’know what I mean? And that is kinda hurtful. And then you want to know why nobody want to believe in the medical system? Yeah like, well. Duh! [211009]

And finally, Benjamin discusses the difference when receiving care from providers who take a harm reduction-oriented approach versus those who do not. In the following passage, Benjamin, a 45-year-old sexual minority Latino man diagnosed 10 years ago, explains how his drug use was identified as problematic in a care setting that did not meet people “where they were at,” compared to the harm reduction program where he sought HIV care and was even able to reinitiate an ART regimen. Similar to participants who face multiple, chronic, and long-term challenges – from homelessness to diabetes to hepatitis C virus – Benjamin’s engagement with HIV care and adherence to ART is greatly affected by his drug use and mental health issues:

Even when I came to New York City, I was doing pretty well, but then I just like after a while, like the regimen got to be so – like, the different pills. Three different, you know. I was like, let me stop it altogether. Plus, you know, I was an addict, you know. Drinking. And I picked up the drug called crack cocaine … So, that right there, you know, it’s not really a good – But they even say, even if you are drugging still keep taking your medications … I was off my medication and I wanted to go back to, you know – and I had left [AIDS Service Organization]. [AIDS Service Organization] was my actual doctor. I left there. I went to [large hospital] and I didn’t really like the providers that were there ‘cause they didn’t really use the (harm reduction) model as to – meeting you where you’re at. And then they said, well, you know, we found cocaine in your system. I’m like okay, so, okay, but, you know, I’m actually using. But then I went back to [AIDS Service Organization], and I was able to disclose a lot of those things about mental health issues. The nurse practitioners, they were able to understand that mental health does play a part in people not taking their HIV medication. It also plays a part in if you’re using. You’re not concerned about taking your medication. So I was able to have part in my care where she put me on Stribild (a one-pill, once-a-day complete ART regimen). [512047]

These seemingly disparate experiences regarding ART initiation and discontinuation share a unifying principle: participants were not strictly bound by one or the other outcome (that is, either engaged in care and taking ART or not engaged and not taking ART), but frequently moved back and forth between the two, thus providing many possibilities for intervention along the way.

## Discussion

Past research has documented the associations among substance use and poor engagement along the HIV care continuum and with adverse HIV health outcomes. In particular, crack cocaine use, heroin use, and poly-substance use are the strongest predictors of ART non-adherence compared to other substances (48–51). However, little research has examined the relationship of substance use to ART initiation, an important but under-studied stage in the HIV care continuum, nor has it captured the mechanisms by which substance use impedes ART initiation and engagement in HIV care from the perspectives of PLWH themselves. Our qualitative study addresses these gaps in the literature by providing a rich description of these phenomena among African-American/Black and Hispanic PLWH who have delayed, declined, or discontinued ART, many of whom were also not well engaged in HIV care. The Theory of Triadic Influence proved useful in drawing attention to social- and structural-level barriers to ART initiation and HIV care, and how they manifest at the individual level, in addition to the individual-level barriers that are most prominent in the literature. Importantly, this study highlights how the problems of ART delay or discontinuation and poor engagement in HIV care cannot be separated from the larger context of poverty and structural violence against/structural racism toward African-American/Black and Hispanic PLWH (17, 44, 52–54). Indeed, substance use problems generally occurred within relations of marginalization grounded in poverty, such as insecure housing, intimate partner violence, lack of employment opportunities, and other forms of deprivation, all of which complicate health-care decisions and access. Furthermore, results highlighted the complex social position of most participants, namely, HIV-infected, poor, African-American/Black or Hispanic, female (in some cases), sexual minority (in some cases), formerly incarcerated (for some), and drug using. This confluence of multiple stigmatized identities shaped participants’ experiences in HIV care and approaches to ART. These findings are consistent with an intersectionality lens, a health disparities framework that posits that social categories, such as “substance user” and “person of color,” are much more than the sum of their parts, but instead are mutually constitutive and interdependent (55, 56), as these study results illustrate. Yet, in the context of a confluence of stigmatized identities in this population, results highlighted the utility of placing substance use in the foreground, because, as the present study highlights, substance use is a primary, visible impediment to ART initiation and HIV care for this group.

Thus, the present study sheds light on the various ways substance use interferes with ART uptake. We found that heavy substance use served as a barrier to ART initiation and HIV care by shifting priorities away from health behavior, reducing confidence in one’s medication management skills, perhaps rightly so in some cases, and triggering depression, which reduced motivation to initiate ART. Furthermore, medical distrust appears to be an important contributing factor. Participants, already wary of ART and ART side effects (57), were in particular fearful of potential interactions between street drugs and ART that might lead to adverse side effects. However, they did not feel they could discuss this concern with providers. Furthermore, providers’ assumptions about drug diversion were an additional set of thorny issues adversely affecting patient–provider relationships, and impeding ART uptake, from the perspectives of PLWH. Thus, the patient–provider interface is an important avenue for intervention. Although studies of patient–provider communication are numerous in the HIV clinic context (32, 58), little is known about strategies to elicit and ameliorate the patients’ fears and providers’ assumptions, some accurate, some false, associated with PLWH’s substance use. In fact, Kreps argues that racial disparities in health outcomes are related to communication problems within the health-care system, which lead to unequal access to health information and inadequate participation in health-care decision-making (59).

### The Non-Linear Path of Managing HIV Infection

Participants’ relationship to ART initiation and their efforts to engage in care rarely followed a linear, predictable path, but rather one marked by varying degrees of ambivalence and uncertainty. Current treatment guidelines recommend that patients initiate ART as a long-term commitment and avoid unscheduled interruptions that lead to drug resistance and increase the chance of treatment failure (37). However, this goal does not seamlessly align with the clinical complexities, competing demands, and pressing social realities, including substance use, faced by African-American/Black and Hispanic PLWH. In fact, in a qualitative study of people who had recently been diagnosed with HIV at an urban public hospital, definitions of what it meant to engage in care by PLWH did not readily match provider definitions; for some participants, their contact with medical and ancillary staff *was* their engagement in care, despite that they might not have been considered active clinic patients and were thus deemed to be disengaged from care (60). This suggests the need to adapt pathways to care that privilege patient’s experiences and the meanings they attach to these processes. Similarly, Muessig and colleagues note that adherence interventions need to better convey adherence as a continuous, changing process, rather than a fixed state (57), and as we found in the present study, ART initiation decisions and behavior, the precursor to adherence, follow this same, changing course.

### The Role of Multiple Stigmas

From the perspectives of PLWH, both their actual and perceived substance use patterns were associated with stigma directed at them from some HIV health-care providers and in other settings they encountered. Furthermore, these experiences of stigma were common impediments to engagement in HIV care and uptake of ART. Indeed, it is well documented that both HIV- and substance use-related stigma are composed of the dimensions of stereotyping, prejudice, and discrimination, are directed toward, and experienced and internalized by PLWH, and this, in turn, can impede engagement in HIV care (13, 43, 61–63). But, participants’ narratives also illustrated that the judgmental stance they experience toward their perceived substance use cannot be disentangled from their socioeconomic status, race/ethnicity, involvement in transactional sex, sexual minority status, and/or histories of incarceration. The Theory of Triadic Influence proved, again, to be well suited to our qualitative investigation. While any one of these factors alone – individual, social, and structural – would reveal important insights about the phenomena under study, it was by unpacking barriers at multiple levels of influence that we begin to observe their complex dynamics.

Thus, as we have noted, the present study highlights how the problems of low uptake of ART and engagement in HIV care emerge from poverty and structural violence (17, 53). While this study raises the question of whether HIV care providers stigmatize PLWH who use substances, it also raises questions regarding what larger structural or societal factors foster those interactions. Indeed, understanding stigma perceived by PLWH to originate from health-care providers and settings cannot be separated from the larger context, which include an overburdened health-care system where providers may be overwhelmed and frustrated by the system in which they operate, particularly as they treat patients with multiple comorbidities and psychosocial needs (64). For example, a recent survey of over 700 HIV clinicians revealed their decisions to delay prescribing ART among substance users even when medically warranted, primarily because of their concerns that patients would lack the stability needed to adhere to the demanding regimen (65). Indeed, in some cases, this concern may be warranted, as participants in this study discussed. On the other hand, in our own past research with HIV primary care providers in NYC, we found that HIV care providers reported considerable challenges balancing a non-judgmental attitude about substance use and promoting better health among their patients, stemming mainly from a lack of training regarding and understanding of substance use and its treatment (66). Indeed, HIV care providers expressed frustration about their inability to address substance use concerns as effectively as they did those associated with HIV. Furthermore, as infectious disease physicians are often the primary providers of HIV care, some reported they had little interest in addressing substance use concerns. This mismatch between providers’ skills and interests and participants’ needs may be experienced as stigma. Thus, providing enhanced substance abuse training to HIV care providers in HIV clinics, as well as ancillary and complementary services and interventions in HIV clinics, has potential to reduce experiences of stigma and thereby improve patients’ engagement in care and treatment outcomes, and possibly improve health-care workers’ experiences of providing HIV care. Our analyses support findings in the literature that show that health-care providers may indeed benefit from further training in substance use and mental health treatment issues (67). Indeed, settings that have integrated substance use treatment, including harm reduction approaches along with HIV medical care, may be especially well suited to provide comprehensive HIV care for PLWH who use drugs (68).

### Managing Substance Use in Order to Initiate ART

Study findings also demonstrate that substance use did not universally pose a barrier to participants’ initiation of ART. Several participants described how they were able to integrate their substance use – whether through harm reduction, abstinence-based treatment, or behavior modification – into their engagement with HIV care, including ART initiation. Access to non-judgmental support through medical and ancillary health providers was a primary mechanism by which participants initiated ART and engaged in HIV care. Participants highlighted how therapists, nurses, case managers, and physicians in some cases were able to engage them in a supportive manner over a period of time, building trust and communication, a model of care that departs from the typically narrow focus on single health interventions to treat a health condition. Participants’ discussion of these experiences foregrounds the significance of compassion, care, respect, and being treated as a “whole person.” This may be a key, if not primary, component of successful ART initiation and adherence, as well as general medical care of HIV-infected individuals who have many cooccurring medical and psychosocial needs. In fact, ancillary service providers may be critical in this respect, given the demands for health-care providers to maintain short appointment times. However, all providers have tremendous power to influence their patients’ decisions, as we highlight above.

### Limitations

One general limitation of this study is that its purposive sampling method may limit its generalizability to African-American/Black and Hispanic PLWH as a whole. Our qualitative sample was, however, drawn from a larger study sample, which reflects several key demographic features present in the HIV/AIDS epidemic in NYC with regards to race, gender, age, socioeconomic status, and years since diagnosis. The purposive, rather than random sampling method, is fitting with the goals of qualitative research, which aims for depth rather than breadth. Despite these limitations, this qualitative study offers insights into the needs and challenges faced by African-American/Black and Hispanic PLWH who have past histories of, or are currently using substances, but who are not equally benefiting from the treatment advances that have brought significant improvements to PLWH’s quality of life and extended its length. Future research will be extended to include the perspective of providers, and to examine policy and structural barriers to good HIV outcomes for this population in greater detail.

### Implications

The present study highlights a number of addressable barriers to ART initiation and engagement in HIV care for this vulnerable population of PLWH, as well as gaps in current practice and potential junctures for intervention efforts. Indeed, study findings suggest the need for interventions at the level of individual PLWH (e.g., to reduce barriers and motivate and support readiness to initiate ART and concomitant changes to substance use patterns when appropriate); health-care providers (e.g., increase skill and comfort to assess and manage substance use among PLWH and understand stigma and its causes and effects); health-care settings (e.g., to supplement the brief health-care encounter with services for substance users including those with the harm reduction approach); and larger organizational and social structures (e.g., stable housing, adequate income support, and the transition out of incarceration).

## Author Contributions

MG conceived of the overall study concept and design and played a primary role in writing the manuscript. RG, RF, and SH planned the data analyses, conducted analyses, and helped draft the manuscript. AK designed study procedures and helped draft the manuscript. ES designed study procedures and directed the study. NL was a study coinvestigator and contributed to study design and concept. AR participated in study design and interpretation of results. CM-P conducted qualitative interviews and consulted on aspects of theory and analysis. NS and HW contributed in the area of the biomedical interface between substance use and HIV care, which fostered data interpretation. CH contributed to data analyses and manuscript preparation. CC was a study coinvestigator and contributed to study design and concept. All authors read and approved the final manuscript.

## Conflict of Interest Statement

The authors declare no potential conflicts of interest with respect to the research, authorship, and/or publication of this article.
